# A High Performance All-Textile Wearable Antenna for Wristband Application

**DOI:** 10.3390/mi14061169

**Published:** 2023-05-31

**Authors:** Asma Ejaz, Iqra Jabeen, Zia Ullah Khan, Akram Alomainy, Khaled Aljaloud, Ali H. Alqahtani, Niamat Hussain, Rifaqat Hussain, Yasar Amin

**Affiliations:** 1 ACTSENA Research Group, Department of Telecommunication Engineering, University of Engineering and Technology, Taxila 47050, Pakistan; 2 Antenna and Electromagnetics Research Group, School of Electronic Engineering and Computer Science, Queen Mary University of London, London SE1 9DE, UK; 3 College of Engineering, Muzahimiyah Branch, King Saud University, Riyadh 11451, Saudi Arabia; 4 Intelligent Mechatronics Engineering, Sejong University, Seoul 05006, Republic of Korea; 5 Independent Researcher, London E1 4NS, UK

**Keywords:** wearable antenna, EBG (Electromagnetic Band Gap), SAR (Specific Absorption Rate), medical applications

## Abstract

A compact, conformal, all-textile wearable antenna is proposed in this paper for the 2.45 GHz ISM (Industrial, Scientific and Medical) band. The integrated design consists of a monopole radiator backed by a 2 × 1 Electromagnetic Band Gap (EBG) array, resulting in a small form factor suitable for wristband applications. An EBG unit cell is optimized to work in the desired operating band, the results of which are further explored to achieve bandwidth maximization via floating EBG ground. A monopole radiator is made to work in association with the EBG layer to produce the resonance in the ISM band with plausible radiation characteristics. The fabricated design is tested for free space performance analysis and subjected to human body loading. The proposed antenna design achieves bandwidth of 2.39 GHz to 2.54 GHz with a compact footprint of 35.4 × 82.4 mm^2^. The experimental investigations reveal that the reported design adequately retains its performance while operating in close proximity to human beings. The presented Specific Absorption Rate (SAR) analysis reveals 0.297 W/kg calculated at 0.5 W input power, which certifies that the proposed antenna is safe for use in wearable devices.

## 1. Introduction

The developments in body area network (BAN) devices have been fueled by the rise in the demand for IoT (Internet of Things) connected devices. Wearable devices have accelerated the digital transformation of several sectors, including health monitoring, tracking, rescue operations, and gaming [1,2]. Wearable antennas enables wireless data transmission between on-body devices and off-body nodes (external node) in BAN for a variety of applications [3]. A seamless data transfer operation over a reliable wireless link is critical, and cannot be guaranteed without integrating a high-performance antenna into the system.

Current technological solutions are not entirely enough to fulfill the demands of pervasive health monitoring in coming era. There is a great demand to scale up the research towards reliable wireless connectivity, smart sensors, wearable devices, and wireless implants [4]. With adoption of modern techniques in healthcare systems, quality healthcare services can be provided to a vast majority of victims with reduced risk and without the inevitable delays encountered in manual checkups [5]. According to a report by the WHO (World Health Organization) [6], climate change, social and technical changes in our lifestyle, and shifting demographic trends are causing a noticeable increase in infectious diseases. Consequently, keeping up the ratio of available medical staff and specialists to the increasing number of patients is becoming problematic [7]. The concept of smart healthcare systems and telemedicine can drastically improve medical services across a region [8,9].

Wearable technology has the potential to revolutionize the lifestyle of the common man. Its tendency to simultaneously become faster and smaller holds out the prospect of a future orbiting around wearables. The trend of consumer preferences reveals an ever-increasing demand for wearable health monitoring devices. Users find self-tracking devices helpful in keeping them engaged with their health [10]. However, when it comes to the collection and processing of medical data, device sensitivity and reliability are extremely critical [11]. These gadgets are expected to provide accurate real-time data for intensive care in hospitals. A similar level of quality and functionality is required in emergency and rescue services. Therefore, it is of immense importance to carefully analyze the trade-offs among cost, size, ease of use, and performance.

### 1.1. Challenges and Related Research

Designing high performance antennas is very simple, provided that ideal conditions are applied. A bulky antenna structure operating in an environment where no interferences exist is all that is needed. Efficient antenna design is obtained when the size is about half the wavelength. This means that an antenna of almost 8 cm is required for the 1900 MHz Global System for Mobile Communications (GSM) band. There should be no interfering electronics nearby when this antenna is operating. This is obviously not possible; hence, researchers continue working to optimize antennas in size and performance as per the required application.

Wearable antennas have become more common in recent years due to their appealing qualities and potential to provide wireless environments and communication while being lightweight, tiny, affordable, and customized. Maintaining the uniformity of features in various environments is one of the main problems encountered in the wearable antenna development, covering aspects such as temperature, humidity, proximity to people and their clothing, wash cycles, etc. [12]. Because most wearable technology is used on clothing, it may be important to create antennas that are simple to incorporate into clothing, such as a textile antenna. These are made of a textile conductive element connected to another textile material serving as a substrate [13]. The performance upon bending the antenna and the amount of radiation absorbed by the human body are the two key issues that designers of wearable antennas should keep in mind. The antenna’s degree of flexibility is important, as it may be positioned in circumstances that call for bending. Moreover, as the antenna is operating close to the body, SAR must be calculated in order to ensure consumer safety [14]. Several methods have been used to reduce the SAR level in order to comply with international standards. Utilizing Artificial Magnetic Conductors (AMCs), sometimes termed Electromagnetic Band Gap (EBG) structures, and High Impedance Surfaces (HISs) are the methods that are most frequently explored; both are placed on the back of wearable antennas to lower the obtained SAR value [15].

Several articles have presented textile wearable antennas [16,17,18] and highlighted the challenges associated with material characterization and fabrication issues. A reflector-backed technique can be adopted in a conventional way, which usually ends in compromised results, especially in terms of bandwidth. However, these on-fabric designs offer improved wearability. Having said this, fabric based antennas are more prone to band shifting due to their inherent material properties [19,20]. Thus, a wider bandwidth is more desirable to account for any band shifting resulting from possible structural deformation.

Designing antenna for on-body operation is challenging from various aspects. The antenna should be compact and conformal to offer a good user experience. Daily life activities should not be interrupted by wireless data transmission. Furthermore, human tissues have high permittivity, which allows for near-field coupling with the body [21]. An antenna operating close to the body suffers from impedance mismatch and reduced radiation efficiency unless accompanied by an isolating material [22]. The literature suggests several techniques to design efficient wearable antennas by employing high-impedance surfaces. These surfaces have been recognized to improve antenna radiation performance while providing shelter from adverse effects for bodily organs near the device. Therefore, meta-surface based wearable antennas have been widely studied [23,24]. In [25], the antenna was isolated from the body by applying an AMC surface of 3 × 3 cells printed on vinyl substrate. While the structure has a small foot print, it offers low gain and low directivity.

A fabric antenna for wearable applications that is low-profile, small, conformal, and has HIS was presented in [26]. Because it imitates the functioning of a Perfect Magnetic Conductor (PMC), which does not exist in nature, the inclusion of HIS in the wearable antenna design offers artificially unique feature. In addition, such a device has the ability to regulate electromagnetic behaviour. The resulting wearable antenna has greatly increased performance in terms of efficiency, FBR (Front to Back Ratio), directivity, and SAR value. To lower the antenna’s SAR value and raise its performance characteristics, an innovative planar UWB antenna with reduced backside radiation using meta-material (MM) was designed on felt textile substrate appropriate for wearable applications [27]. MMs are man-made engineering designs with two special advantageous qualities that do not occur naturally. First, they have capacity to control the propagation of electromagnetic waves with a zero-phase shift, such as those from AMC. Second, they possesses an electromagnetic band gap (EBG) property that can block surface waves in all directions [28]. In [29], by integrating rigid and flexible substrates, the authors concentrated on proposing a high gain, high efficiency, and low SAR antenna. In this way, the antenna’s seamless operation and good performance were ensured by printing of a folded-ring radiator on a robust low-loss substrate. To ensure wearer comfort, a ground plane made of conductive cloth was attached to the textile substrate, raising the broadside gain and considerably lowering the SAR. Numerous wearable antennas with good isolation and wide bandwidth have been reported to date. In [30], a proximity-fed textile microstrip patch antenna was proposed for wearable applications for the first time, resulting in increased bandwidth and good radiation efficiency. Adopting a proximity feed instead of alternative feeding techniques increases the antenna’s operational bandwidth while reducing the design’s size, which makes it better suited for integration into wearable systems. With the recent rapid advancement in communication technologies, the field of wireless body area networks (WBAN) has expanded significantly [31]. It now supports a wide range of applications, including individualized health care systems, patient monitoring systems, rescue systems, battlefield survival, and wearable gaming consoles. Several designs have been proposed and evaluated in the open literature for suitability as wearable antenna elements, including cavity-backed [32], microstrip [33], inverted-F [34], planar [35], and vertical monopole [36] antennas. However, all these antennae offer limited bandwidth, a sizable footprint, and a high front-to-back ratio, and they protrude from the body. Alternately, when electromagnetic band gap (EBG) structures are incorporated into wearable antenna designs, they can provide a high degree of isolation from the human body and decrease the SAR value [24,37]. The innovative structures of metasurfaces are frequently employed in antenna applications such as frequency and electronically scanned antennas and leaky wave array antennas. Additionally, these structures can enclose tiny radiation sources in order to improve their directivity at particular resonance frequencies. To considerably advance the state-of-the-art designs, a high-performance miniaturized EBG backed monopole antenna working in the MBAN band was proposed in [38]. The authors focused on miniaturization of a cutting-edge EBG unit cell, resulting in an array reflector made up of 2 × 2 unit cells, and integrated a monopole antenna at a later stage for performance improvement. However, the study lacked system-level validation of antenna performance, and was solely concerned with fine-tuning the design parameters.

### 1.2. The Proposed Work

The development of the antenna design proposed in this work was carried out with the aim of obtaining a compact footprint and reduced geometrical complications to ensure good fabrication on textiles while ensuring that the design is able to offer maximum user comfort. These objectives were met by designing the integrated antenna on a denim substrate with a compact footprint. An EBG unit cell is set to operate in the ISM band, with a 2 × 1 EBG array is selected for the sake of a miniaturized configuration. When the number of unit cells is limited by size constraints, the usual approach of placing an EBG array behind an antenna operating at the same frequency ends with inferior performance in terms of bandwidth and radiation efficiency. For this reason, a novel approach utilizing a 2 × 1 metasurface with a floating ground plane is adopted. This approach allows for a more compact structure with enhanced performance. A similar kind of a wearable antenna was presented in [39] for the medical body area network band, in which the superiority of the aforesaid approach was verified using a 2 × 2 metasurface array. However, the bandwidth of the structure presented in that paper was limited, and the design was barely bendable owing to a thicker non-flexible substrate layer. Therefore, in this work we investigated an exclusive unit cell configuration for bandwidth enhancement, which was then exploited to achieve the desired results in terms of reduced dimensions while maintaining an all-textile approach.

## 2. Antenna Design Methodology

Because the ground plane of the EBG structure is displaced, the design methodology is quite different from the conventional approach [40], which begins with antenna design followed by metasurface development and its integration. Here, the EBG unit cell is initially designed and studied to provide an optimum bandwidth with the center at 2.45 GHz. A 2 × 1 metasurface is formed, then the antenna is optimized such that the integrated design is resonant at the zero-phase reflection frequency of the EBG structure.

### 2.1. EBG Formation

**Figure 1:**
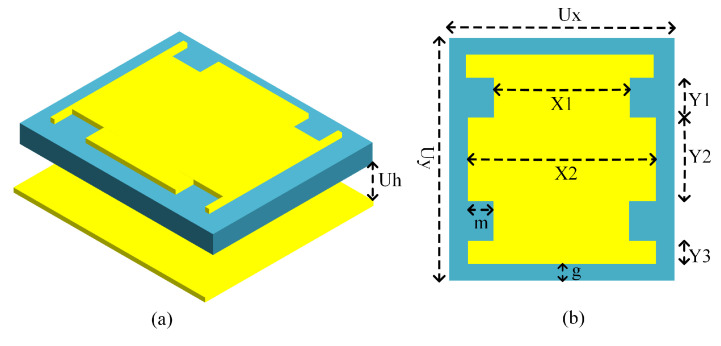
The proposed unit cell design: (**a**) 3D view, where Uh = 5 mm; (**b**) front view, where Ux = 37 mm, *Uy* = 39.5 mm, *Y1* = 7 mm, *Y2* = 16.3 mm, *Y3* = 2.6 mm, *X1* = 23, *X2* = 33, *g* = 2 mm, *m* = 5 mm.

**Figure 2:**
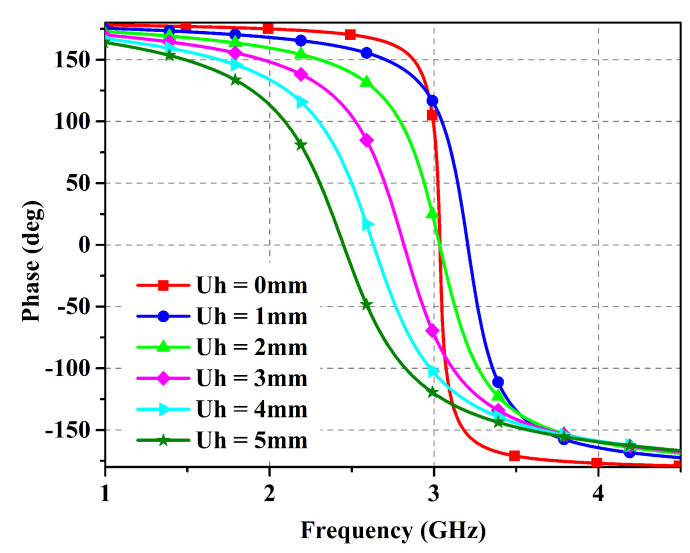
Reflection phase of the unit cell.

**Figure 3:**
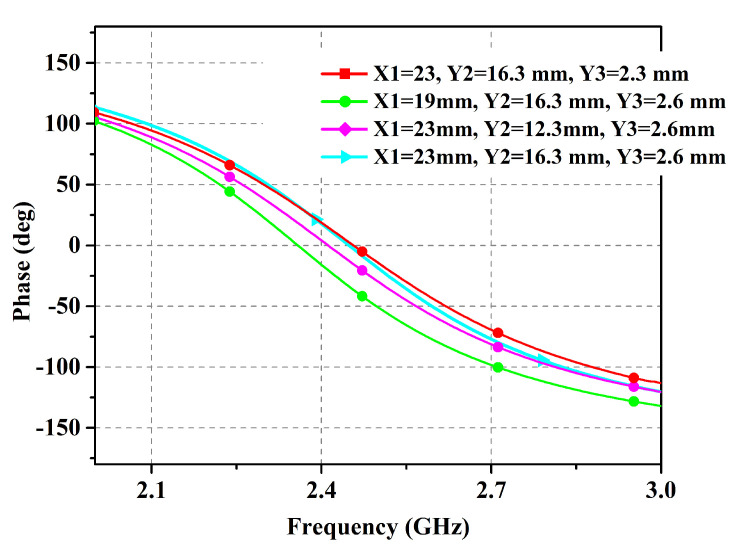
Parametric analysis of the unit cell design.

With the objective of reducing the number of adjustable variables in the design, a simple slotted-square EBG unit cell was tuned to operate in the desired band using a denim substrate ($\varepsilon_{r}$ = 1.7, δ = 0.02, height = 1.57 mm). Figure 1 shows the unit cell design details along with the dimensions used in the integrated structure. In consideration of the fact that the antenna must necessarily suffer from a detuning effect when placed on the body, a wide impedance bandwidth is preferred to ensure that the resulting shift in resonant frequency can be tolerated. The study of the EBG unit cell by simulations in CST Microwave Studio is revealed in Figure 2. It is evident from the presented results that the bandwidth is improved by moving the ground of the proposed EBG unit cell further away. Thus, the EBG unit cell was reoptimized with ground placed at d = 4 mm, resulting in reduced planar dimensions and enhanced impedance bandwidth. It is noteworthy here that the bandwidth parameter can be successfully controlled using only one variable for the distance. However, the current dimensions are restricted in light of the application area requirements. Another parametric analysis of the design dimensions is depicted in Figure 3 for the proposed unit cell. It shows the movement of the resonance frequency of the unit cell when changing the variables *X1*, *Y2*, and *Y3*. The resonance frequency is recorded in the zero-degree reflection phase, and is required to be set at 2.45 GHz. It is observed that by increasing or decreasing the values of the design variables the resonance frequency is moved to either a higher or lower spectrum. However, there is very little impact on the bandwidth. Therefore, the bandwidth offered by the unit cell is mainly controlled by the *Uh* parameter. The other design parameters control the shifting of the resonance frequency.

### 2.2. The Proposed Integrated Design

**Figure 4:**
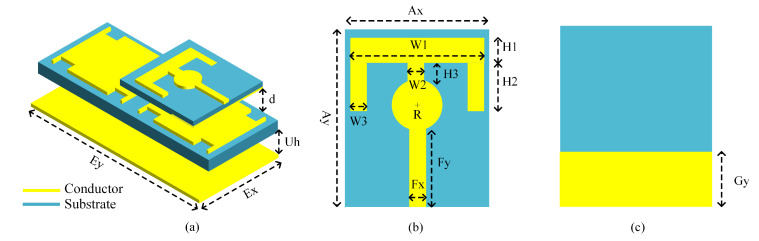
The proposed wearable antenna design: (**a**) 3D view with dimensions *Ex* = 82.4 mm, *Ey* = 35.4 mm, *Uh* = 5, *d* = 1.9 mm; (**b**) front view, where *Ax* = 35 mm, *Ay* = 52 mm, *W1* = 28 mm, *W2* = *W3* = 4 mm. *H1* = 7 mm, *H2* = 13 mm, *H3* = 6.42 mm, *R* = 5 mm, *Fx* = 2.5 mm, *Fy* = 26.12 mm; (**c**) back view, where *Gy* = 10 mm.

**Figure 5:**
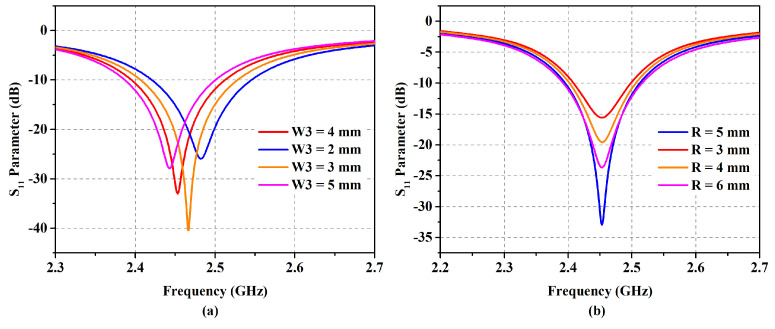
Parametric analysis of the proposed design for variables *W3* (**a**) and *R* (**b**).

**Figure 6:**
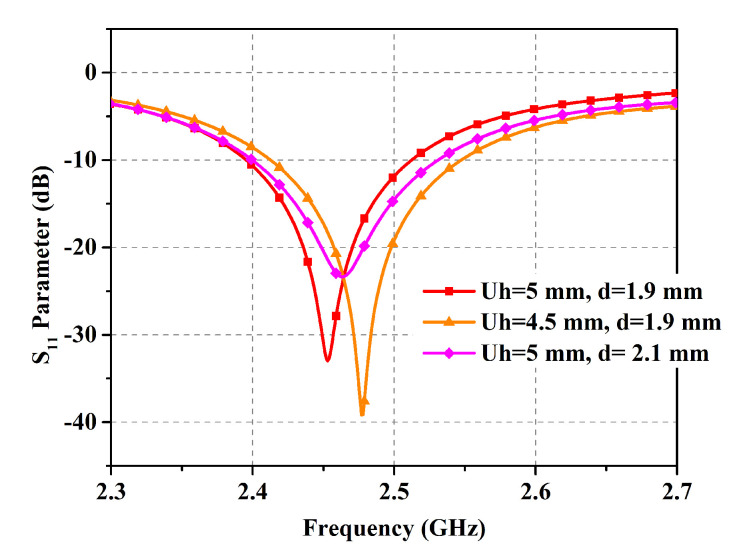
Parametric analysis of the proposed design for variables *Uh* and *d*.

With the developed compact 2 × 1 EBG surface, a planar monopole antenna is placed above it at a close distance. The spacing allows for electromagnetic coupling; thus, the EBG surface plays a role in controlling the emitted radiations as well. The antenna dimensions are optimized to produce resonance at 2.45 GHz. The final integrated design with dimensions is presented in Figure 4. The parametric analysis of the proposed embedded design is manifested in Figure 5, where it is observed that the radius of the center circle controls the antenna impedance match owing to the fact that the feedline is directly connected to it. Similarly, frequency shift can be administered by the tuning variable *W3*. The parametric analysis of the integrated design for the distance between the antenna radiator and EBG substrate (*Uh*) and EBG ground plane displacement (*d*) is shown in Figure 6. Reducing the distance between the antenna radiator and the EBG substrate by 0.5 mm shifts the resonance to a higher frequency. Similarly, increasing the variable *d* by 0.2 mm causes a frequency shift to a higher spectrum and reduces impedance matching. These values are optimized to achieve the desired resonance frequency with optimum band coverage.

## 3. Results and Discussion

In this section, we present the simulation and experimental analysis of the proposed wearable antenna. The prototype development involves conductive fabric and readily available denim fabric, achieving an all-textile solution to wearable devices.

The accuracy and manufacturing efficiency of low-cost wearable antenna designs are determined by fabrication procedures. Wet-etching, screen printing, ink-jet printing, and embroidery processes are the most widely used ways of wearable fabrication reported in the literature [41,42,43]. These methods can be utilized to fabricate antennas that are long-lasting, affordable, and comfortable for users to wear on a daily basis. Many electronics manufacturers employ screen printing because it is an easy and affordable strategy. There are limitations of the screen-printing method, however, including a low number of realizable layers, lack of conductive layer thickness control, and poor printing resolution. Inkjet printing is one of the most reasonably priced printing methods; however, the primary issue with this technique is the incompatibility of certain conductive ink types due to their greater particle size, which can cause nozzle blockage. Though embroidered antennas are widely regarded as ideal replacements for conventional antennas in flexible electronics, they have drawbacks compared to antennas made of metallic materials. For example, an embroidered geometry is much more stretchable than metallic antenna sensors on inlays. The limited resolution of the yarn stitches and the impact of stretching render delicate geometries useless [44,45].

**Figure 7:**
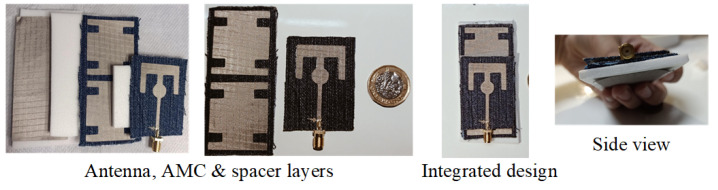
Views of the fabricated prototype.

**Figure 8:**
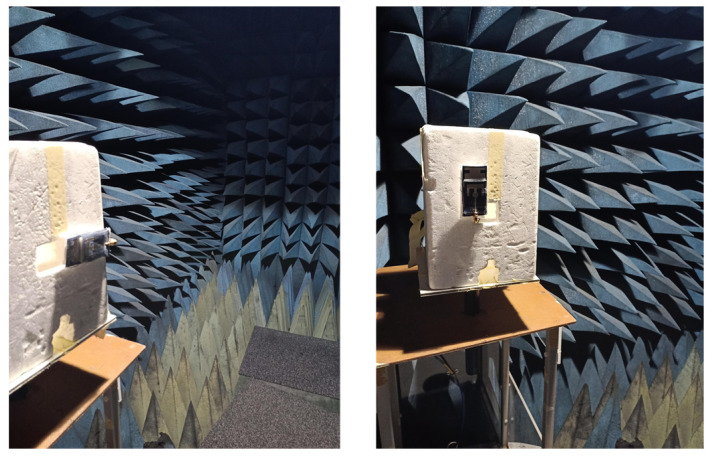
Measurement setup.

The fabricated antenna is shown in Figure 7, with different views used to present the entire design morphology. To characterize the dielectric properties of the available denim substrate, a split-cylinder resonator (Agilent 85072A, Keysight Technologies, Inc., Santa Rosa, CA, USA)) and Keysight material characterisation software (N1500A-003 Materials Measurement Suite 2015) were used. Shieldex Nora Dell metallized fabric constitutes the conductive part of the design. It is worth mentioning here that executing a fabric-based design fabrication with high accuracy is complicated. Therefore, a Silhouette Cameo machine from the Materials Engineering Laboratory at Queen Mary University of London was used for precise fabric cutting. The machine is able to cut the fabric with high accuracy (fabrication tolerance varying from 0.1 mm to 0.5 mm). Spacers are required to fill in the gaps between different layers of the structure. Rohacell foam was used for this purpose, as its dielectric constant is close to that of air and does not affect the performance. A 50 Ω SMA connector was soldered to the monopole feedline with the help of silver conductive epoxy. To validate the simulated results, the developed prototype was tested for various performance parameters, as shown in Figure 8. The measurement setup involved a vector network analyzer to obtain reflection coefficient. Radiation characteristics were experimentally measured inside an anechoic chamber, where antenna mount setup was adjusted to have line-of-sight alignment with the transmitting horn antenna.

### 3.1. Free-Space Simulation and Measurement Results

**Figure 9:**
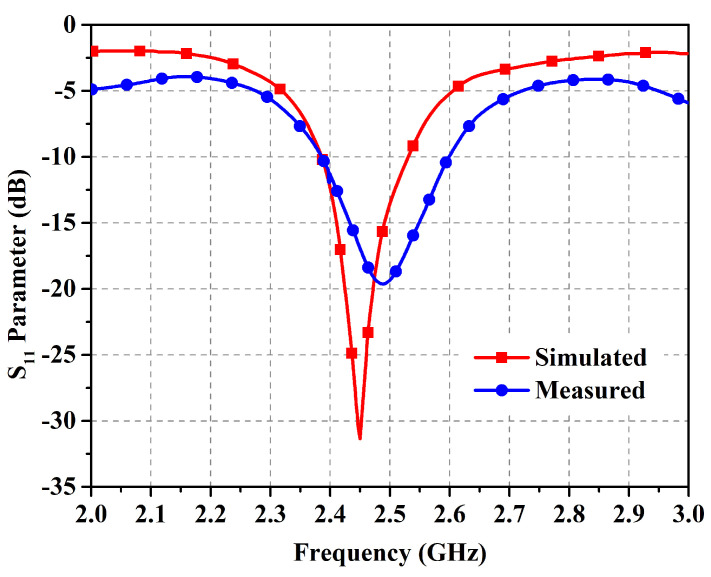
Simulated and measured reflection coefficient (in decibels) in free space.

**Figure 10:**
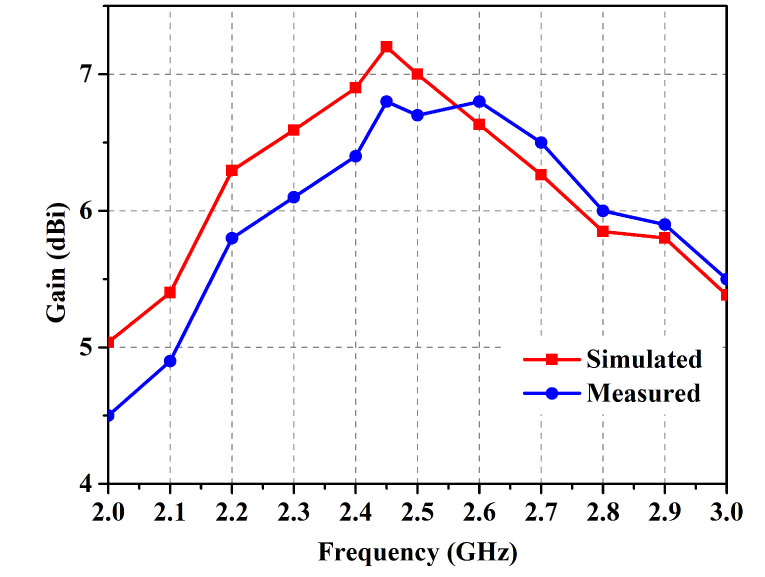
Simulated and measured gain in free space.

**Figure 11:**
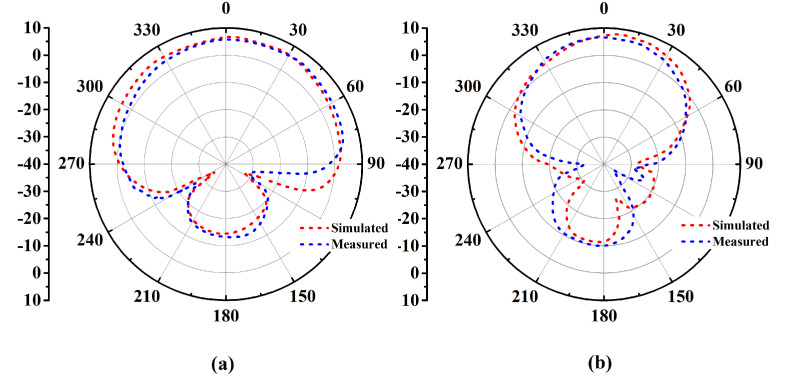
Free space simulated and measured radiation patterns (Degree vs. dB) in (**a**) the E-plane and (**b**) the H-plane.

The antenna prototype was experimentally analyzed for the performance parameters of reflection coefficient (shown in Figure 9), gain (shown in Figure 10), and radiation patterns (shown in Figure 11) in the two orthogonal planes. The analysis reveals close correspondence between simulated and measured results. The minor discrepancies are due to fabrication imprecision as well as to non-ideal uniformity of the separation between the metasurface layer and the antenna. The reflection coefficient vs. frequency curve exposes a −10 dB bandwidth, ranging from 2.39 GHz to 2.54 GHz with the center at 2.45 GHz, providing generous coverage of the ISM band. The fractional bandwidth comes out to 6.1%.

### 3.2. On-Body Simulation and Measurement Results

Free space measurement results predict admirable performance of the proposed wearable antenna. However, it is important to analyze the performance variations in the wristband case. The fact that the human body consists of lossy tissues affects the antenna’s efficacy. The electrical properties of human organs are distinct, making them suitable EM (electromagnetic) energy absorbing materials. Thus, the radiated energy of the antenna is subject to being partially absorbed by the user’s body. The amount of this effect depends on several parameters, including the distance between the antenna and the user and the radiation characteristics of the antenna. The EM wave loses energy upon interaction with a dielectric medium. The human body is a non-homogenous dielectric medium that is characterized by the μ, ϵ, σ, and loss tangent (tan δ). The body is composed of various tissue layers, and each layer has different electrical properties.

**Figure 12:**
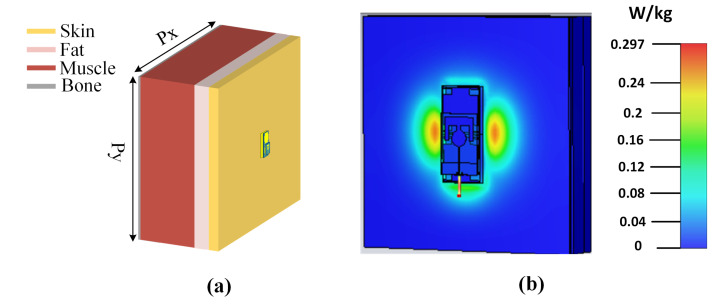
(**a**) Layered phantom model with dimensions Px = Py = 400 mm and (**b**) SAR evaluation.

There are different ways to develop a human body model in a simulation tool to observe on-body performance. There are human anatomical models available in EM simulation tools, such as the voxel phantom model, which can accurately describe the human anatomy. These models are more rigorous and realistic than the multilayered phantom model; however, they require substantial computational resources. For analysis based on simulations, we constructed a multi-layered phantom model in the simulation tool. The dielectric constant, loss tangent, and mass density of each layer were defined as per the reported data from the literature. The model shown in Figure 12a mimics a real human body. Thus, the proposed antenna was mounted above this phantom model. The accuracy of on-body simulation results can be increased using voxel models. However, this requires large computational power. Due to limited availability of computational resources, the finite size layered phantom model was used in our simulations. The phantom model dimensions should be kept large enough to ensure that the phantom edges are at least λ/4 distance away from the antenna edges. Because the on-body measurement results are provided, the ambiguity in the simulation results can be ignored.

**Figure 13:**
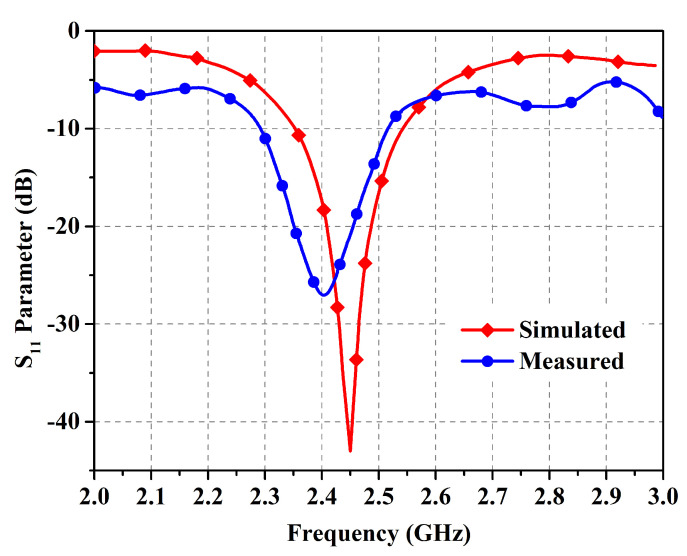
Simulated and measured reflection coefficients of the antenna (in decibels) when worn on the body.

**Figure 14:**
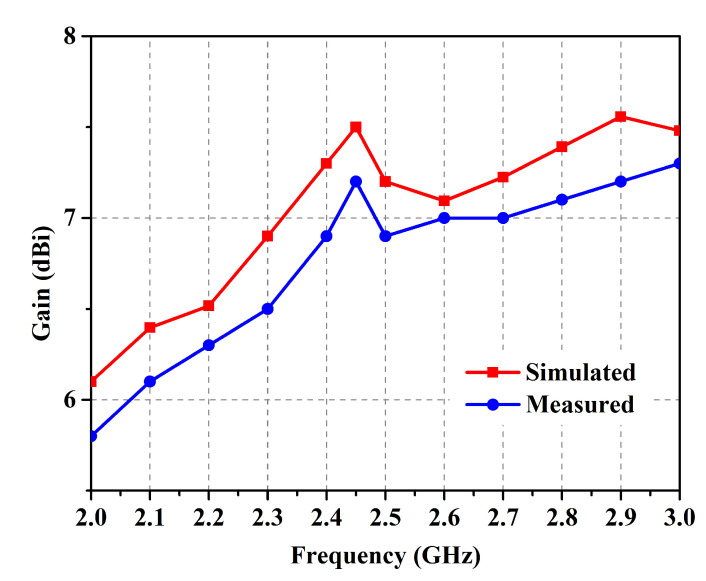
Simulated and measured gain of the antenna when worn on the body.

**Figure 15:**
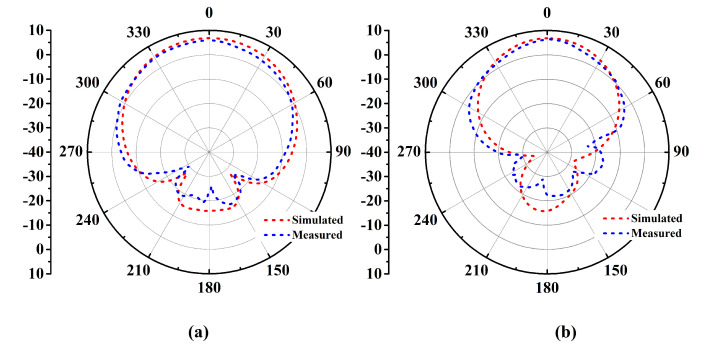
On-body simulated and measured radiation patterns of the antenna (degree vs. dB) in (**a**) the E-plane (**b**) the H-plane.

The human body-loaded antenna performance parameters of the reflection coefficient (in decibel), gain, and radiation pattern are depicted in Figure 13, Figure 14 and Figure 15, respectively. Gain is observed to be improved when more power is directed upwards, leaving behind less radiation power to be absorbed by the underlying body. The back lobe of the radiation pattern is further suppressed due to reflections from the high permittivity phantom layers.

In addition to the above-mentioned analysis, the presented design operation was further investigated by mounting the antenna over the wrist of the body. The recorded results are presented in Figure 13. The low impact of the human body on impedance matching is due to the shielding property of the metasurface layer. The analysis shows that the impedance match is slightly different for each body organ. Nevertheless, our antenna successfully operates in the required band for on-wrist analysis. Moreover, the size of the design is appropriate for being embedded in a wrist band.

### 3.3. SAR Evaluation

When the human body is exposed to EM radiation, one result is elevated body temperature [46]. Regulatory standards are defined to ensure that this exposure remains within safe limits. One guideline is that proposed by the IEEE and ICNIRP, according to which occupational and public exposure is limited to 10 and 2 W/kg, respectively. Occupational exposure is termed controlled exposure, in which a person is exposed to radiation as a part of their job. Professionals are made aware of threats and can control their exposure by leaving a room where radiation is present or by other means taught to them in training sessions and safety programs. Such places are equipped with warning signs and labels to bring awareness. Exposure can be of a transient nature, when a person moves through a location with high radiation. Public exposure, on the other hand, involves uncontrolled exposure in which people have no control over their exposure. As the subjects are unaware of the potential threat, more restrictive limits are applied in this scenario. All consumer devices follow the restrictions of the latter category.

In addition to the requirements of enhanced performance parameters of wearable antennas, arguably one of the most critical concerns for any device operating close to the human body is its impact on human health. This matter is assessed by SAR analysis, which indicates the amount of radiation absorbed by human tissues. The standard limitations are to be followed by all wearable devices. Thus, the SAR value should be less than 1.6 W/kg for 1 g of tissue, a limit set by the FCC. SAR dissemination is calculated for the diverse layers of human tissues. It is obvious from Figure 12b that the SAR value is far below the defined threshold. This value is calculated at 0.5 W input power and 1 mm distance between the antenna and the phantom model. Hence, the proposed all-textile antenna can be safely used in any wearable application.

A comparison between the proposed wearable antenna and works published previously in the literature is provided in Table 1. It is worth mentioning here that [30] reports a 2 × 1 EBG backed antenna with a compact footprint. However, the substrate is not suitable for wearable applications owing to its rigidness. The proposed work incorporates a floating-ground based EBG to achieve enhanced bandwidth of the antenna while utilizing a unit cell array of only 2 × 1.

## 4. Conclusions

In this paper, we propose a novel flexible wearable antenna exploiting a floating-ground based EBG to enhance antenna performance. The achieved bandwidth is able to account for possible band shifting under bending or human body loading effects. The antenna was fabricated using Shieldex Nora Dell metallized fabric over a denim substrate layer. Cutting, pasting, and alignment of the design was carried out using A Silhouette Cameo machine. The computed overall far-field properties of the antenna indicate good performance with adequate gain and efficiency. An SAR analysis is presented, and the results validate the appropriateness of the suggested embedded design for wearable devices. 

## Figures and Tables

**Table 1:** Comparison with previously published works.

References	Size (mm^2^)	Substrate	Reflector	No of Unit Cells	Gain (dB)
[47]	42 × 30	Felt	EBG	4 × 3	6
[48]	40 × 20	Textile	EBG	2 × 1	4.48
[49]	124 × 124	Rogers RO3003	AMC	4 × 4	8.4
[50]	50 × 50	Latex	AMC	3 × 3	1.81
[14]	45 × 45	Fabric	HIS	3 × 3	7.47
[51]	30 × 20	Denim	-	-	2.05
[5]	145 × 112	Denim	HIS	4 × 3	6.19
[52]	120 × 120	Leather and textile	EBG	3 × 3	7.98
[53]	81 × 81	Felt	AMC	3 × 3	7.3
[54]	60 × 60	Fabric	EBG	2 × 2	6.45
The proposed work	35.4 × 82.4	Denim	EBG	2 × 1	7.46

## Data Availability

Not applicable.
